# VIBE: an R-package for VIsualization of Bulk RNA Expression data for therapeutic targeting and disease stratification

**DOI:** 10.3389/fonc.2024.1441133

**Published:** 2025-01-29

**Authors:** Indu Khatri, Saskia D. van Asten, Leandro F. Moreno, Brandon W. Higgs, Christiaan Klijn, Francis Blokzijl, Iris C. R. M. Kolder

**Affiliations:** ^1^ Translational Data Science, Genmab, Utrecht, Netherlands; ^2^ Translational Data Science, Genmab, Princeton, NJ, United States; ^3^ Target Discovery, Genmab, Utrecht, Netherlands; ^4^ Discovery Data Science, Genmab, Utrecht, Netherlands

**Keywords:** antibody, disease stratification, visualization, oncology, R package, targeted therapy, transcriptomics, bioinformatics tool

## Abstract

**Background:**

Development of cancer treatments such as antibody-based therapy relies on several factors across the drug-target axis, including the specificity of target expression and characterization of downstream signaling pathways. While existing tools for analyzing and visualizing transcriptomic data offer evaluation of individual gene-level expression, they lack a comprehensive assessment of pathway-guided analysis, relevant for single- and dual-targeting therapeutics. Here, we introduce VIBE (VIsualization of Bulk RNA Expression data), an R package which provides a thorough exploration of both individual and combined gene expression, supplemented by pathway-guided analyses. VIBE’s versatility proves pivotal for disease stratification and therapeutic targeting in cancer and other diseases.

**Results:**

VIBE offers a wide array of functions that streamline the visualization and analysis of transcriptomic data for single- and dual-targeting therapies. Its intuitive interface allows users to evaluate the expression of target genes and their associated pathways across various cancer indications, aiding in target and disease prioritization. Metadata, such as treatment or number of prior lines of therapy, can be easily incorporated to refine the identification of patient cohorts hypothesized to derive benefit from a given drug. We demonstrate how VIBE can be used to assist in indication selection and target identification in three user case studies using both simulated and real-world data. VIBE integrates statistics in all graphics, enabling data-informed decision-making.

**Conclusions:**

VIBE facilitates detailed visualization of individual and cohort-level summaries such as concordant or discordant expression of two genes or pathways. Such analyses can help to prioritize disease indications that are amenable to treatment strategies such as bispecific or monoclonal antibody therapies. With this tool, researchers can enhance indication selection and potentially accelerate the development of novel targeted therapies with the goal of precision, personalization, and ensuring treatments align with an individual patient’s disease state across a spectrum of disorders. Explore VIBE’s full capabilities using the vignettes on the GitLab repository (https://gitlab.com/genmab-public/vibe **).**

## Introduction

The success of many therapeutic strategies hinges on the accurate targeting of pathological sites, specific cell types or biological mechanisms within the body (1). Evaluation of target expression is pivotal to ensure that the therapeutic agents precisely address the malfunctioning cells or proteins, thereby minimizing collateral damage to healthy tissues (2). A prime example of precision targeting is immunotherapy, where the inherent capacity of antibodies to bind antigens with high specificity is leveraged to target specific cells. Antibody therapy provides a precise method for treating diverse disease types including hematological and solid cancers, HIV, and autoimmune-related diseases (3–6). Antibodies, composed of two antigen-specific Fab regions and an Fc region interacting with immune components (7), facilitate diverse therapeutic effects based on either agonizing or antagonizing a target, such as direct tumor growth inhibition, apoptosis induction, and/or recruitment or inhibition of immune cells. Genetic engineering broadens these functionalities, for example, by enabling dual-targeting antibodies that bind two distinct antigens, either on the same or different cells. One example of this strategy is the therapy Epcoritamab, a bispecific antibody recently approved for relapse or refractory (R/R) diffuse large B-cell lymphoma, which binds CD20 on tumor cells and CD3 on T cells, enhancing T cell-mediated tumor kill (8–10).

Evaluating the mRNA expression of target genes to identify indications with high levels of target expression is a strategy to select promising drug candidates and prioritize indications for further study. In addition to the target genes, a multitude of other genes – particularly those involved in downstream signaling pathways of the target gene(s) – may significantly influence the therapeutic efficacy (11). Therefore, mRNA co-expression between transcripts can elucidate the underlying molecular mechanisms and potential resistance pathways within coregulated patterns, thereby informing the design of more effective combination strategies (12–14).

Several existing tools facilitate the visualization of expression at the individual gene level. For example, TCGAplot (15) and GEPIA (16) provide visualizations for single genes and their correlations to immune-related genes, but they lack capabilities for visualizing gene pairs or implementing expression thresholds for prioritization. Likewise, cBioPortal (17) offers visualizations for individual targets alongside disease stratification across various clinical parameters, but it does not accommodate the visualization of gene pairs, pathways, or user-defined groups. Devis (18), GENAVi (19), and SEQUIN (20) focus on RNASeq data processing and visualization of differentially expressed genes, yet they do not support the exploration of multiple genes or pathways for target prioritization and patient stratification. Thus, there exists a need for a novel tool that provides the functionalities absent in existing platforms. Such a tool would be indispensable for advancing the field of targeted antibody therapies by facilitating a deeper understanding of inter-gene relationships and pathway dynamics.

Here, we present VIBE, an R package uniquely tailored for the VIsualization of Bulk RNA Expression data applicable to technologies such as bulk RNA-seq, EdgeSeq, and microarray technologies. With VIBE, researchers can delve into the expression of specific genes or gene pairs within large transcriptomic datasets, such as the publicly available TCGA (21), GTEx (22, 23) or XENA (24) databases, as well as custom datasets. Moreover, VIBE provides averaged scores for gene sets, such as pathway-associated genes, informing a comprehensive overview of gene interactions and functions within the pathway of interest. VIBE simplifies the characterization and visualization of target and pathway expression in specific cancer types or subtypes.

## Methods

The package offers a wide range of functions, each with customizable parameters. All VIBE visualizations are generated with the data-visualization package ggplot2 (25), which can easily be adjusted to individual requirements with additional ggplot2 commands. Detailed vignettes are available on the GitLab page. A dummy data set is included upon installation to enable out-of-the-box exploration of VIBE’s functionalities.

### Generating a simulated data set

The simulated data was generated using the rnorm (12) package with mean and standard deviation for 35 genes (including “Tumor target” and “Immune target”) for 16 solid tumor types (**Table 1**) pre- and post-treatment. The Stringi (12) package was used to randomly generate the patient IDs and sample IDs. The patient IDs were generated to match pre- and post-treatment data. For every tumor type, the “dummy” log_2_ transcripts per million (TPM) values were randomly generated for 100 patients, 50 each for pre- and post-treatment samples (**Table 1**). Users can define categories based on their research question. To illustrate this process, an additional parameter was added such that patients were assigned to two different databases (database1 or database2) as an example.

**Table 1:** Description of VIBE’s simulated dummy dataset.

Indication	Abbreviated indication	Paired pre- and post-treatment samples	Unmatched database samples	
			database1	database2
Bladder cancer	BLCA	50	52	48
Breast cancer	BRCA	50	100	0
Cervical cancer	CERV	50	50	50
Colorectal cancer	CRC	50	62	38
Head and neck squamous cell Carcinoma	HNSCC	50	50	50
Neuroendocrine tumors	NET	50	50	50
Non-small lung cancer	NSCLC	50	0	100
Ovarian cancer	OV	50	56	44
Pancreatic adenocarcinoma	PDAC	50	36	64
Prostate cancer	PRAD	50	44	56
Renal cell carcinoma	RCC	50	38	62
Sarcoma	SARC	50	54	46
Small cell lung cancer	SCLC	50	50	50
Stomach adenocarcinoma	STAD	50	64	36
Triple-negative breast cancer	TNBC	50	50	50
Uterine carcinoma	UTEN	50	54	46

### Harmonizing the expression datasets

**Figure 1:**
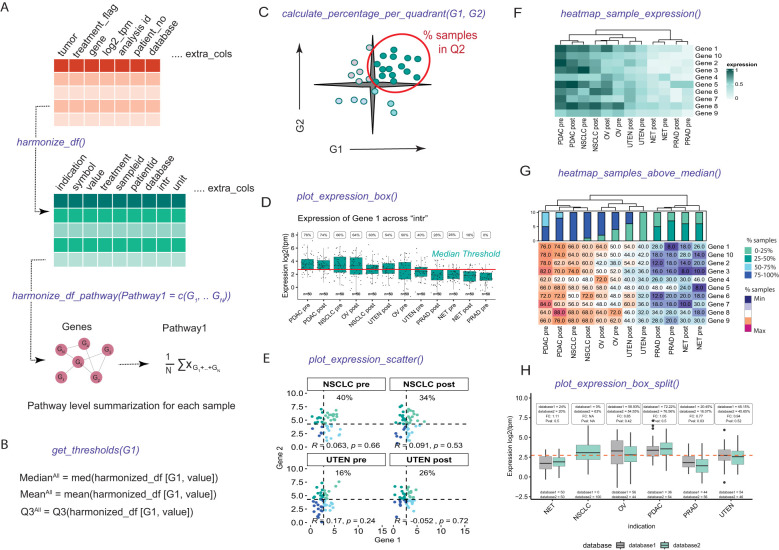
Implementation and overview of the statistics and visualization capabilities of the VIBE package. **(A)** The df_harmonize() function harmonizes dummy data, creating a structured dataframe with appropriate column names and format for VIBE functionalities. The harmonize_df_pathway() function combines genes into pathways for VIBE visuals. **(B)** The get_thresholds() function defines gene-specific thresholds (mean, median or 75% quantile (Q3)) for expression analysis and indication selection. **(C)** The calculate_percentage_per_quadrant() function extends get_thresholds() for dual-gene analysis, displaying percentage of samples in quadrants, correlation, and statistical significance (Spearman correlation). **(D)** plot_expression_box() presents a boxplot with sample distribution, thresholds, percentage satisfying thresholds, and user-defined grouping variables and groups (i.e. “intr”) generated when harmonizing the dataframe. **(E)** plot_expression_scatter() plots a scatterplot with user-selected grouping variables and groups and statistics calculated by calculate_percentage_per_quadrant() function. **(F)** heatmap_sample_expression() visualizes gene expression via heatmaps, allowing grouping variable selection, group plots, gene splitting, and ordering of genes. **(G)** heatmap_samples_above_median() represent the percentage of samples satisfying threshold for all the genes with similar functionalities as mentioned in heatmap_sample_expression(). The barplots above the heatmap depict the distribution of samples across four quartiles based on percentage: Q1 (0–25%), Q2 (25–50%), Q3 (50–75%), and Q4 (75–100%). The indications are clustered using Hierarchical clustering, as indicated by the dendrograms. **(H)** plot_expression_box_split() function visualizes the expression of Gene 1 grouped by additional grouping columns with statistics (% above median for each group, the fold change (FC) and Kruskal-Wallis p-value (Pval)) above.

The expression dataset for VIBE should consist of the following essential columns: i) patient ID, ii) sample ID, iii) gene or feature name, iv) expression values, v) the unit used for plotting captions, and vi) grouping or plotting columns such as indication and treatment. To ensure the dataset is structured optimally for analysis and visualization, VIBE offers the *harmonize_df()* function. This function harmonizes the dataset by updating column names and generates additional columns that serve as grouping variables for statistical analysis or visualization purposes (**Figure 1A**). Users have the flexibility to choose which additional columns to retain, enabling them to create multiple comparative visualization schemes tailored to their specific research needs.

In line with the pathway-supported decision-making process, VIBE provides the *harmonize_df_pathway()* function. This function offers users the capability to define a list of genes representing gene signatures or pathways. Utilizing this information, the function calculates the average expression values of all the genes within each gene signature or pathway. The *harmonize_df_pathway()* function then returns a structured data frame that is ready for further visualization using VIBE’s functionalities.

### Defining thresholds

Rather than imposing arbitrary thresholds for identifying indications or classifying samples into high or low-expression bins, our methodology allows users to pre-define thresholds based on the mean, median, or 75% quantile. This is achieved using the *get_threshold()* function in VIBE (**Figure 1B**). For consistency throughout this manuscript, we have chosen the median as the threshold.

To calculate thresholds, the median mRNA expression of each selected gene was computed separately for all categories (Median^category^). Subsequently, the median expression of all samples (Median^All^) was determined to dichotomize the category binarily. Specifically, if Median^category^ was greater than Median^All^, the category is considered to be of interest for the new therapeutic. Additionally, the percentage of samples per category per dataset was calculated where Median^category^ is greater than Median^All^. This approach allows for a flexible and user-defined thresholding strategy, enabling meaningful analysis and comparisons across different datasets and indications.

### Analysis of gene associations and gene-pathway interactions

One of the distinctive features that set VIBE apart from other applications is its capacity to visualize complex interactions and correlations between multiple genes or pathways simultaneously. For composite assessments of two genes or pathways, VIBE utilizes the previously generated thresholds to classify samples into four quadrants based on the expression levels (e.g., high/high, high/low, low/high, low/low) of the selected genes or pathways. Users have the flexibility to choose any quadrant for visualization and can use the *calculate_percentage_per_quadrant()* function to calculate the percentage of samples within the chosen quadrant (**Figure 1C**). Additionally, VIBE calculates the Spearman or Pearson correlation depending on user input between the selected genes or pathways, providing insights into their potential interactions and associations.

For composite assessment of antibodies versus multiple genes or pathways, VIBE calculates the percentage of samples within the chosen quadrant for each comparison. These results can be effectively visualized in a heatmap, allowing users to easily discern and interpret patterns and trends across various comparisons.

### Statistical analysis and visualization

The visualization capabilities of the VIBE package extend beyond basic graphics, enabling researchers to draw meaningful statistical inferences directly from the plots. The visuals are extended using ggplot2 (12) and ComplexHeatmap (12) functionalities. The *plot_expression_box()* function, for instance, provides a boxplot that incorporates the threshold and percentage of samples satisfying the threshold within the plot itself. This eliminates the need for a separate table and facilitates the selection of user-defined groups (**Figure 1D**). Additionally, the *plot_expression_scatter()* function offers a scatterplot between two genes, where quadrant-specific colors are defined by the thresholds of the two genes. This scatterplot not only visualizes the percentage of samples in the selected quadrant but also displays the correlation between the two genes and its statistical significance within the given dataset using Spearman correlation (**Figure 1E**). The functions provided also enable the plotting of specific indication categories. To ensure statistical robustness, the thresholds for these categories are calculated using the complete dataset. Visualizations are then generated specifically for the categories chosen by the user.

For a comprehensive overview of relevant genes in the dataset, VIBE offers the heatmap representation of gene expression in user-defined groups (**Figure 1F**). Moreover, the heatmap also shows the percentage of samples that are above the user-defined threshold, aiding researchers in identifying significant gene expression patterns (**Figure 1G**). Furthermore, the *plot_expression_box_split()* function accommodates additional grouping variables, as an extension of *plot_expression_box()* with statistics (% above median for each group, the fold change (FC) and Kruskal-Wallis p-value (Pval)) depicted above (**Figure 1H**). This function allows users to compare the expression of a gene in control versus treatment conditions, providing valuable insights into the impact of different conditions on gene expression.

VIBE offers additional advanced visualization and statistical inferences for comprehensive genomic data analysis. Demonstrated in its vignette and results section, VIBE enables researchers to identify indications of interest, assess gene and pathway correlations, and make data-driven decisions, enhancing precision in drug development and disease research.

## Results

The robust capabilities of VIBE are demonstrated here through three case studies. In the first case study, the statistical and visual representations are leveraged to effectively identify cancer indications of interest for a hypothetical antibody-drug conjugate (ADC). In the second case study, VIBE’s comprehensive pathway and gene signature analyses are used to showcase how researchers can assess the potential impact of multiple pathways in tandem with target expression. The third use case leverages publicly available RNA-seq datasets to uncover potential therapeutic targets in melanoma patients who progressed on nivolumab using VIBE. Together, these three use cases highlight how VIBE can provide valuable support in making well-informed decisions regarding therapeutic interventions, enhancing the precision and effectiveness of treatment strategies.

### VIBE as a tool for identifying indications of interest for a hypothetical antibody-drug conjugate (Case study 1)

**Figure 2:**
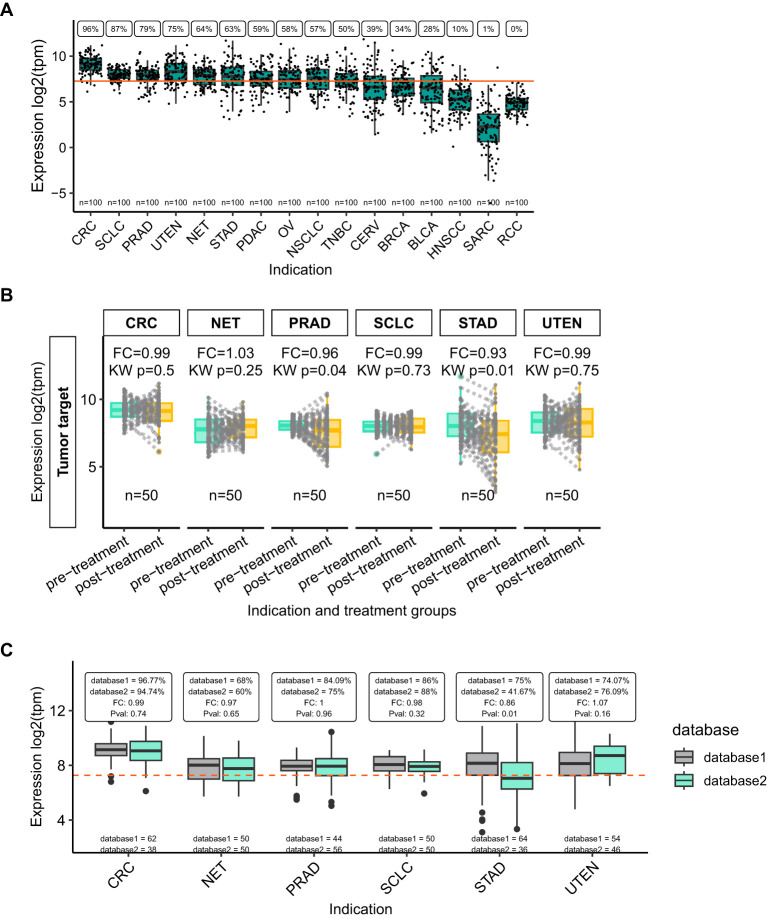
“Tumor target” expression highlights indications of interest for a novel ADC. **(A)** VIBE’s plot_expression_box function shows a box for each group of interest, indication in this example, showing simulated data included in the VIBE package. Dots indicate individual samples; the orange line indicates the median expression of the plotted gene across all samples in the dataset. The percentage of samples with higher than median expression is printed above each box, while the number of samples in each group is shown just above the x-axis. **(B)** The plot_box_pre_post function plots a box for pre- and post-treatment samples, with each dot representing a sample, while lines connect paired samples. The fold change (FC) and Kruskal-Wallis p-value (KW p) are printed above each group of interest. **(C)** The plot_expression_box_split function allows for the comparison of non-matched data and includes the fold change (FC) and Kruskal-Wallis p-value (Pval) between the two groups. The number of samples included in each dataset is printed just above the x-axis. The orange dotted-line indicates the median expression for this gene across all samples in the combined dataset. Indication abbreviations are listed in **Table 1**.

In this first case study, VIBE was used in a hypothetical scenario to identify potential cancer indications of interest for an ADC directed against the ‘Tumor target’ gene. In the dummy dataset, high expression levels of the “Tumor target” gene is important for drug-target engagement and informing both the pharmacodynamics and potential efficacy for this targeting strategy. The “Tumor target” expression levels were visualized using boxplots across the cancer types revealing that expression was the highest in CRC, SCLC, PRAD, UTEN, NET and STAD (**Table 1**). These cancer types may therefore be considered as potential candidates for the ADC therapy (**Figure 2A**). However, these findings should still be confirmed using single-cell approaches to determine whether the “Tumor target” gene is indeed highly expressed by cancer cells, and not healthy (immune) cells. In addition, protein expression in the top-ranking tumor types should be confirmed in further experiments, for example by immunohistochemistry.

In clinical trials evaluating novel oncological agents, the patient cohort frequently comprises individuals who have previously undergone one or more treatments. Consequently, it is imperative to analyze and compare the expression levels of the drug’s target in samples collected before and after treatment. This approach is critical for understanding the therapeutic’s potential efficacy and mechanism of action within a treatment-experienced population. VIBE’s specialized function *plot_box_pre_post()* plots paired pre- and post-treatment samples and calculates the fold-change and p-value between the two states. Using this functionality in the dummy dataset, STAD was identified as the tumor type where the “Tumor target” expression was lower in post-treatment samples compared to pre-treatment samples (**Figure 2B**). This indicates that the ADC therapy could be less effective in post-treatment STAD patients compared to pre-treatment patients.

VIBE allows for the inclusion of additional labels or groups of interest with unmatched samples in the dataset. To exemplify such an analysis, the dummy data was processed using VIBE’s *harmonize_df()* function while keeping the additional column representing unmatched samples from “database1” and “database2” (**Figure 1A**). VIBE’s function *plot_expression_box_split()* allows comparison of gene expression differences between the unmatched samples. In the dummy data, the “Tumor target” expression was lower in STAD samples in database 2 compared to database 1 (**Figure 2C**), indicating that the underlying data for this indication warrants further investigation. The *plot_expression_box_split()* function can also be used to compare tumor to normal samples e.g. GTEx vs TCGA in a real-world setting.

### VIBE as a tool for pathway-guided indication selection for a novel monoclonal or bispecific antibody (Case study 2)

In this case study, an analysis involving more than two genes or pathways was conducted. Depending on the intended mechanism of action (MoA), the efficacy of a novel therapy may depend on the (co)-expression of multiple genes or even entire pathways. Consequently, high expression of specific genes and pathways may inform therapy-enhancing hypotheses. As an example, this case study aims to select cancer indications for a bispecific antibody targeting both tumor (“Tumor target”) and immune (“Immune target”) cells. This bispecific antibody’s MoA promotes immune cell-tumor cell interaction, leading to immune cell activation, and resulting in tumor cell kill by the immune cell.

To exemplify the capabilities of VIBE, we used the “Immune target” and “Tumor target” variables from the dummy dataset to visualize targets from a bispecific antibody therapy. Only the indications with a median expression higher than the threshold for the “Tumor target” (Case study 1) were selected for further analysis.

**Figure 3:**
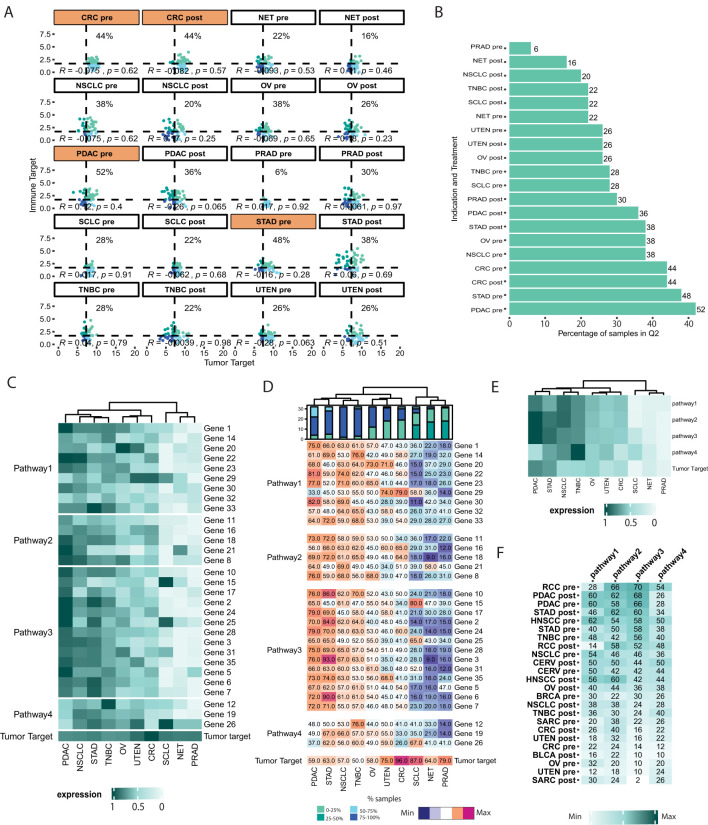
Identification of indications for bi-specific or pathway-guided single ADC. **(A)** Scatterplot representing the distribution of samples in 4 different quadrants based on the user-defined threshold (“Tumor target” vs “Immune target”). VIBE’s *plot_expression_scatter()* function generates a flow cytometry-like scatterplot, dividing samples into four quadrants based on the median expression of “Tumor target” and “Immune target” across all samples. The *plot_groups* argument of the function was used to select the indications as shown in **Figure 1A**. Indications and treatment groups with over 40% of samples expressing high levels of both “Tumor target” and “Immune target” are highlighted in orange. **(B)** Boxplot representing the percentage of samples in user-defined groups and quadrants. The *plot_perc_pop()* function in VIBE generates a boxplot that displays the percentage of samples expressing high levels of both “Tumor target” and “Immune target”. **(C)** Heatmap representing the expression of user-defined grouping of genes (pathways) in user-defined/selected indications. VIBE’s *heatmap_sample_expression()* function is based on the ComplexHeatmap package (12), enabling easy grouping of genes into pathways and visualization of their expression along with the target of interest, such as the “Tumor Target” in this case. One can easily visualize the change in the expression of genes in pathways compared to the “Tumor target”. **(D)** Heatmap representing the percentage of samples in user-defined grouping of genes (pathways) and user-defined/selected indications. VIBE’s *heatmap_samples_above_median()* function, also based on ComplexHeatmap (12), allows users to visualize the percentage of samples with high expression levels of individual genes within the defined pathways. **(E)** Heatmap representing the expression of pathways in user-defined/selected indications. VIBE’s *heatmap_sample_expression()* function allows users to visualize the average expression of genes in pathways in conjunction with the “Tumor Target”. Comparing the composite pathway expression to individual genes, as shown in **Figure 3C**, offers a more robust approach for making decisions on indication selection, particularly when considering the mode of the ADC’s mechanism. **(F)** Composite analysis of pathways vs ADC. VIBE’s *heatmap_composite_scores()* function generates a heatmap representing the percentage of samples in the Q2 quadrant of the scatterplot. The function allows the user to define a threshold to select indications based on the scores. In this case, a threshold of 20 was used.

Of note, these threshold values continue to be calculated based on the entire dataset to ensure statistical validity and robustness. By examining the percentage of samples falling within the second quadrant of the scatterplot (**Figure 3A**) and the corresponding bar plot (**Figure 3B**), we identified PDAC pre-treatment, STAD pre-treatment, CRC pre-treatment, and CRC post-treatment as potential indications and treatment groups of interest for the bispecific antibody.

The evaluation of pathways is crucial in assessing the effectiveness of antibodies with an MoA that is associated with modulation of multiple canonical biological processes and plays a pivotal role in the selection of suitable indications and treatment groups. For instance, in scenarios where mRNA signatures such as T cell infiltration or activation are associated with an antibody therapy MoA, high expression of genes in these pathways is essential to represent an inflamed, or “hot tumor”. VIBE offers two distinct scenarios for pathway assessment: (1) evaluating individual genes within a pathway, and (2) composite analysis of pathways using summarized expression of signature genes. To represent the first scenario, heatmap visualizations were used to illustrate gene expression (**Figure 3C**) and the percentage of samples with expression above the median threshold (**Figure 3D**). In the second scenario, averaged expression of genes to represent pathways, were visualized alongside the “Tumor target” in a heatmap (**Figure 3E**). Using these visuals, PDAC and STAD emerged as relevant indications for further investigation.

Although scatterplots, as shown in **Figure 2A**, offer detailed insights into quadrant-specific sample distributions, they become complex when comparing one target with multiple genes or pathways. To address this, the *heatmap_composite_score()* function presents a user-defined quadrant-specific percentage of samples in a heatmap (**Figure 3F**). This allows users to quickly assess and identify patient groups of interest. For example, NSCLC pre-treatment and PDAC pre-treatment patient cohorts showed high expression of both the “Tumor target” and Pathways 1, 2, and 3, (e.g. T-cell activation, T-cell exhaustion or T-cell infiltration) making them potentially relevant indications. On the other hand, SCLC pre- and post-treatment patient cohorts would be of interest, showcasing high expression of the “Tumor target” and pathway 4 (e.g. NK cell signature). Users can flexibly choose the pathways and targets to be represented in the analysis.

This comprehensive approach allows researchers to gain a deeper understanding of the interactions and functional significance of gene pathways, facilitating informed indication selection for therapeutic antibody development. In summary, VIBE’s comprehensive visualization capabilities enable researchers to explore and compare complex interactions between the drug’s target gene(s) and multiple genes or pathways, aiding effectively in the selection of potential indications for therapy development.

### VIBE as a tool for prioritizing potential therapeutic targets in melanoma patients refractory to nivolumab (Case study 3)

To demonstrate the application of VIBE on real-world data, we employed a publicly available melanoma dataset (GSE91061) to identify potential antibody therapy targets for patients who progressed on Nivolumab (26). This dataset includes 51 samples collected before Nivolumab treatment and 58 samples taken after treatment from 65 patients, complete with clinical outcome information (complete or partial responders (CR/PR), progressive disease (PD), and stable disease (SD)). The authors identified 2,670 differentially expressed genes (DEGs) between pre- and on-therapy samples of responders and non-responders (26). 81 DEGs associated with stromal, tumor, and host-immunity pathways were highlighted as being of the most significance. Of these DEGs, we focused on 31 genes known to be expressed on the cell surface (27, 28), identifying these as potential antibody therapy targets. Additionally, we manually curated a list of genes indicative of the presence of CD3 cells, CD8 cells, Tregs (29), T cell activation (30), T cell infiltration (31), T cell exhaustion (29), and T cell cytotoxicity [Tc1, Tc2, Tc3 and Tc4] (32) each evaluated individually (**Supplementary Table 1**).

**Figure 4:**
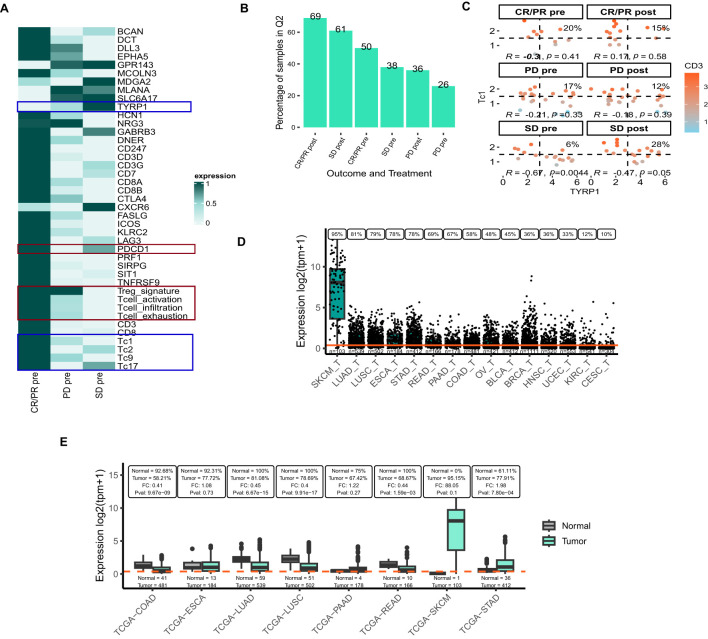
Target prioritization in Nivolumab-experienced melanoma samples using publicly available datasets **(A)** Heatmap representing the expression of the selected genes and user-defined signatures in response and outcome categories at baseline in the GSE91061 dataset. **(B)** Boxplot representing the percentage of samples with high expression of *PDCD1* and *CD3* in response and outcome categories in GSE91061 dataset. **(C)** Flow-cytometry-based scatter-plot representing scatter of *TYRP1* and Tc1 where the samples are colored based on the expression of CD3. The correlation and P-value are reported in the scatterplot along with the %samples in Q2 quadrant. Panels **A-C** include abbreviations: CR/PR, complete response/partial response; PD, progressive disease; SD, stable disease. **(D)** Expression of *TYRP1* across tumor samples from selected TCGA projects, visualized using VIBE’s *plot_expression_box*() function. **(E)** Comparison of *TYRP1* expression between tumor and adjacent normal tissues using the *plot_expression_box_split*() function. At the top of the plot the percentage of samples with *TYRP1* expression above the overall median expression is indicated for both normal and tumor samples in each TCGA-project. The fold-change (FC) and Kruskal-Wallis p-value (Pval) between the tumor and adjacent normal tissues are indicated here as well. In panels **(D, E)**, the orange line indicates the median expression for this gene across all samples in the dataset. The number of samples is printed just above the x-axis. Panels **D, E** include the following TCGA-project abbreviations: SKCM, skin cutaneous melanoma; LUAD, lung adenocarcinoma; LUSC, lung squamous cell carcinoma; ESCA, esophageal carcinoma; STAD, stomach adenocarcinoma; READ, rectum adenocarcinoma; PAAD, pancreatic adenocarcinoma; COAD, colon adenocarcinoma; OV, ovarian serous cystadenocarcinoma; BLCA, bladder urothelial carcinoma; BRCA, breast invasive carcinoma; HNSC, head and neck squamous cell carcinoma; UCEC, uterine Corpus Endometrial Carcinoma; KIRC, kidney renal clear cell carcinoma; CESC, cervical squamous cell carcinoma and endocervical adenocarcinoma.

The expression of the selected genes and T-cell signatures was visualized using a heatmap across response and treatment categories at baseline (**Figure 4A**). *PDCD1*, *CD3*, and the Treg signature were overexpressed in patients exhibiting a CR/PR as compared to SD and PD. Similarly, **Figure 4B** shows that a high percentage of samples, both pre- and post-treatment, with above-median expression levels of *PDCD1* and *CD3* were observed in patients exhibiting CR/PR indicating that these markers may be predictive of positive treatment outcomes on nivolumab.

Conversely, we identified several genes with elevated expression in patients with PD and SD when compared to CR/PR (**Figure 4A**), notably Tyrosinase-related protein-1 (*TYRP1*) and markers associated with cytotoxic T cells (Tc1, Tc2, Tc9, and Tc17). TYRP1 is a transmembrane glycoprotein that is specifically expressed in melanocytes and melanoma cells. Preclinical data suggest that monoclonal antibodies targeting *TYRP1* confer anti-melanoma activity (33). Additionally, the potential of *TYRP1* as a therapeutic target is being explored in a clinical trial testing RO7293583, an investigational drug for TYRP1-positive unresectable metastatic melanomas (NCT04551352). RO7293583 (anti-TYRP1/CD3 T-cell engager) binds to both CD3 on cytotoxic T lymphocytes (CTLs) and TYRP1 found on *TYRP1*-expressing tumor cells. In line with this, we assessed the percentage of samples with high expression of *TYRP1* and Tc1 cytotoxic T cell signature in the GSE91061 dataset (**Figure 4C**). While 28% of post-treatment SD samples had a relatively high expression of both *TYRP1* and the Tc1 signature, overall, the correlation between the two was non-significant or negative across the different outcome groups. To further explore the broad applicability of an anti-TYRP1/CD3 bi-specific across various cancer types we leverage data from the publicly available TCGA dataset (21). *TYRP1* expression was highest in SKCM, followed by LUAD, and LUSC (**Figure 4D**). Yet, the proportion of samples exhibiting high levels of both *TYRP1* and *CD3D* samples was comparatively low in SKCM relative to the lung cancers (**Supplementary Figure 1**). This observation suggests that the targeting strategy in melanoma with an anti-TYRP1/CD3 bispecific modality might be challenging. Nevertheless, the expression of *TYRP1* in tumors was lower for both LUAD and LUSC compared to adjacent normal tissue, implying a lack of tumor specificity of this gene in these tumor types (**Figure 4E**). For SKCM, this comparison could not be adequately assessed due to the lack of normal samples (n=1). In conclusion, the VIBE analysis conducted on the GSE91061 and TCGA datasets suggests that *TYRP1* holds potential as a therapeutic target for melanoma. However, the use of a TYRP1/CD3 bi-specific antibody may not represent the most effective strategy for targeting this malignancy.

## Discussion

The VIBE package is a new visualization tool for developing hypotheses around disease stratification and targeted therapy. This tool facilitates composite analyses of multiple genes or pathways with user-defined thresholds, enabling researchers to understand gene expression dynamics comprehensively. This insight is instrumental for making informed and data-driven decisions in antibody development and targeted therapeutic design.

Our demonstration of VIBE’s functionalities, utilizing both simulated data and publicly available datasets, underscores its value in stratifying cancer types and identifying novel antibody targets. This is particularly relevant in oncology, where the precision in indication selection can significantly impact therapeutic success rates. It is also useful in dissecting drug resistance mechanisms —a prevalent challenge in cancer treatment— by highlighting gene expression and pathway alterations associated with drug resistance to develop strategies for overcoming treatment challenges. Moreover, its ability to represent intricate genes and pathways interactions makes it valuable for systems biology research by providing insights into broader regulatory networks and systems. In essence, VIBE offers insights into gene expression dynamics across vital pathways, with broad implications for advancing personalized treatments and understanding disease intricacies. In VIBE, we utilized classical statistical measures such as fold change and p-value, focusing on the task of indication selection. This approach, aimed at swiftly visualizing therapeutic targets, is designed to expedite decision-making in early drug development by emphasizing target potential over extensive statistical validation.

While tools such as DESeq (34) for differential expression and ReactomeGSA (35) for pathway analysis are available, they lack VIBE’s specialized focus on detailed indication selection. VIBE permits manual gene selection for pathway analysis, offering user-driven flexibility and transparency, particularly for oncology researchers who demand a more tailored approach. In case study 3, we applied VIBE to analyze DEGs found by other tools, showing the expression of these genes and gene signatures across outcome groups and TCGA indications, thereby identifying *TYRP1* as a potential target for melanoma patients who have progressed on nivolumab. In addition, this analysis suggested that a CD3 bi-specific antibody may not be the optimal therapeutic modality due to relatively low expression levels of the underlying genes in the tumor microenvironment, but other options such as *TYRP1* CAR-therapy may prove viable (36). This approach positions VIBE as a complementary addition to the existing suite of genomic data analysis tools, addressing a unique niche in the field.

For future development of the VIBE package, we aim to introduce enhanced features that will expand its analytical capabilities and user experience, including the integration of additional statistical methods and user interface improvements. Simultaneously, we plan to foster community engagement through the establishment of an open-source development platform and user forums. This will not only facilitate valuable feedback and collaborative improvements but also ensure that VIBE evolves in response to the real-world needs of researchers, thereby strengthening its role as a crucial tool in genomic analysis and targeted therapy research.

Overall, the VIBE package distinguishes itself by focusing on the niche of indication selection in the domain of gene expression data analysis. This tool not only integrates statistics in visualizations for single-gene exploration but also provides comprehensive visualizations tailored for pathway-guided single- and dual-targeting antibodies. The visuals including expression levels of target gene(s), genes corresponding to the related MoA and the statistics are instrumental in making data-driven decisions in the indication identification and patient stratification process. These capabilities position VIBE as a relevant resource in the field of oncology, facilitating the drug development pipeline by simplifying the processes of indication selection and patient stratification.

## Data Availability

The simulated data used in this study are included as part of the VIBE R package (https://gitlab.com/genmab-public/vibe/-/blob/main/data/VIBE_data.rda). The melanoma RNA-seq data from Riaz et al. is available at the Gene Expression Omnibus data repository (GSE91061) (26) and can be downloaded at https://www.ncbi.nlm.nih.gov/geo/query/acc.cgi?acc=GSE91061. TCGA tumor and adjacent normal RNA-seq data are downloaded using TCGAbiolinks (21, 37–39). The Rmd notebooks are available via GitLab (https://gitlab.com/genmab-public/vibe/-/tree/main/vignettes).

## Supplementary material

Supplementary Table 1 Genes corresponding to the signatures used for visualizing pathways.

Supplementary Figure 1
*TYRP1*, *CD3D* and *CD3E* expression in selected TCGA projects. The scatterplots show the distribution of samples in four quadrants based on the median expression level of *TYRP1* and *CD3D*. The samples are colored based on the expression level of *CD3E*. Expression levels are reported in log2(TPM+1). The scatterplot also reports the spearman correlation and p-value along with the percentage of samples in Q2 quadrant. COAD, colon adenocarcinoma; ESCA, esophageal carcinoma; LUAD, lung adenocarcinoma; LUSC, lung squamous cell carcinoma; PAAD, Pancreatic adenocarcinoma; READ, rectum adenocarcinoma; SKCM, skin cutaneous melanoma; STAD, stomach adenocarcinoma.
