# Supplementary material for: VIBE: an R-package for VIsualization of Bulk RNA Expression data for therapeutic targeting and disease stratification
Source: Front Oncol. 2025 Jan 29;14:1441133. doi: 10.3389/fonc.2024.1441133 (PMC11815282; doi:10.3389/fonc.2024.1441133)
Supplement: Supplementary Table 1 — Genes corresponding to the signatures used for visualizing pathways. [file DataSheet1.pdf]

VIBE: An R-package for VIsualization of Bulk RNA Expression data for therapeutic targeting and disease stratification

Indu Khatri[1]*, Saskia D van Asten[1]*, Leandro F. Moreno[1], Brandon W Higgs[2], Christiaan Klijn[3,4], Francis Blokzijl[3 †], Iris CRM Kolder[1†]

[1] Translational Data Science, Genmab, Utrecht, Netherlands

[2]Translational Data Science, Genmab, Princeton NJ, USA

[3]Discovery Data Science, Genmab, Utrecht, Netherlands

[4]Target Discovery, Genmab, Utrecht, Netherlands

* These authors contributed equally to this work and share first authorship

[†] Co-corresponding authors

Email: FRBL@genmab.com and IKO@genmab.com

Table S1: Genes corresponding to the signatures used for visualizing pathways.

**Signatures**	**Genes corresponding to signatures**
CD3 cells	*CD3D, CD3E, CD3G*
CD8 cells	*CD8A, CD8B*
Tregs	*CD69, ENTPD1, FOXO1, FOXP3, IKZF4, IL10, IL10RA, IRF4, JAK1, NT5E, PRDM1, SMAD2, SMAD3, STAT3, STAT5A, TGFB1, TGFBR1, TGFBR2, TYK2*
T cell activation	*IL2RA, TFRC, DPP4, CD27, CD28, TNFRSF8, CD40LG, TNFRSF4*
T cell infiltration	*CD2, CD247, CD28, CD3D, CD3G, CD6, GPR171, GZMK, ICOS, ITK, KLRB1, PYHIN1, TIGIT, TRAT1, TRBC1, CD4, CD8A, CD8B*
T cell exhaustion	*B3GAT1, BATF, CASP3, CCL3/L1, CD160, CD244, CD86, CTLA4, EOMES, FASLG, HAVCR2, IRF4, KLRG1, LAG3, LILRB4, MDFIC, PDCD1, PDPN, PROCR, PTGER2, TIGIT, TNFRSF9, TNFSF9, TOX*
T cell cytotoxicity; Tc1	*TNF, INFG, IL2, CXCR3, TBX21*
T cell cytotoxicity; Tc2	*IL4, IL5, CCR4, GATA3*
T cell cytotoxicity; Tc3	*IL9, IL10, IRF4*
T cell cytotoxicity; Tc4	*CCR6, KLRB1, IL17, IRF4, RORC*

Figure S1

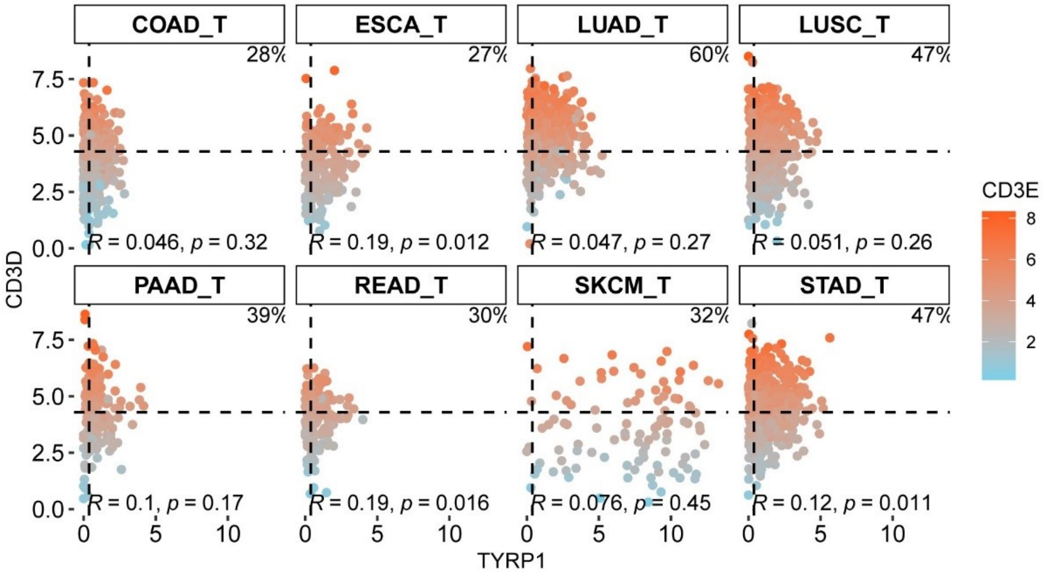

Figure S1: *TYRP1*, *CD3D* and *CD3E* expression in selected TCGA projects. The scatterplots show the distribution of samples in four quadrants based on the median expression level of *TYRP1* and *CD3D*. The samples are colored based on the expression level of *CD3E*. Expression levels are reported in log2(TPM+1). The scatterplot also reports the spearman correlation and p-value along with the percentage of samples in Q2 quadrant. COAD = colon adenocarcinoma, ESCA = esophageal carcinoma, LUAD = lung adenocarcinoma, LUSC = lung squamous cell carcinoma, PAAD = Pancreatic adenocarcinoma, READ = rectum adenocarcinoma, SKCM = skin cutaneous melanoma, STAD = stomach adenocarcinoma.
